# Lung Cancer Chemopreventive Activity of Patulin Isolated from *Penicillium vulpinum*

**DOI:** 10.3390/molecules23030636

**Published:** 2018-03-12

**Authors:** Aymeric Monteillier, Pierre-Marie Allard, Katia Gindro, Jean-Luc Wolfender, Muriel Cuendet

**Affiliations:** 1 School of Pharmaceutical Sciences, University of Geneva, University of Lausanne, 1 Rue Michel-Servet, CH-1211 Geneva 4, Switzerland; aymeric.monteillier@unige.ch (A.M.); pierre-marie.allard@unige.ch (P.-M.A.); jean-luc.wolfender@unige.ch (J.-L.W.); 2 Mycology and Biotechnology group, Plant, Agroscope, Route de Duillier 60, P.O. Box 1012, 1260 Nyon, Switzerland; katia.gindro@agroscope.admin.ch

**Keywords:** cancer chemoprevention, NF-κB, patulin, *Penicillium vulpinum*, Wnt

## Abstract

Lung cancer is the most lethal form of cancer in the world. Its development often involves an overactivation of the nuclear factor kappa B (NF-κB) pathway, leading to increased cell proliferation, survival, mobility, and a decrease in apoptosis. Therefore, NF-κB inhibitors are actively sought after for both cancer chemoprevention and therapy, and fungi represent an interesting unexplored reservoir for such molecules. The aim of the present work was to find naturally occurring lung cancer chemopreventive compounds by investigating the metabolites of *Penicillium vulpinum*, a fungus that grows naturally on dung. *Penicillium vulpinum* was cultivated in Potato Dextrose Broth and extracted with ethyl acetate. Bioassay-guided fractionation of this extract was performed by measuring NF-κB activity using a HEK293 cell line transfected with an NF-κB-driven luciferase reporter gene. The mycotoxin patulin was identified as a nanomolar inhibitor of TNF-α-induced NF-κB activity. Immunocytochemistry and Western blot analyses revealed that its mechanism of action involved an inhibition of p65 nuclear translocation and was independent from the NF-κB inhibitor α (IκBα) degradation process. Enhancing its interest in lung cancer chemoprevention, patulin also exhibited antiproliferative, proapoptotic, and antimigration effects on human lung adenocarcinoma cells through inhibition of the Wnt pathway.

## 1. Introduction

Lung cancer is the leading cause of cancer death worldwide, with 1.69 million deaths in 2015. The high mortality rates are mostly due to late diagnosis, mainly occurring at metastatic stages. Tobacco smoke represents by far the main risk factor for lung cancer and directly accounted for 1.175 million deaths by respiratory tract cancers in 2015 [1]. Among the numerous other environmental risk factors for this cancer, the most important are exposure to ambient particulate matter air pollution (283,000 deaths), asbestos (155,000 deaths), and household air pollution from solid fuels (149,000 deaths). These data enlighten the preventability of this disease, the first lung cancer preventive measure being tobacco control. Despite important efforts in this domain, notably illustrated by the WHO Framework Convention on Tobacco Control and their impact on global smoking prevalence [2], the total number of deaths continues to rise because of increasing population numbers and ageing [1]. Therefore, developing both early diagnosis and lung cancer chemoprevention strategies would complement tobacco control efforts and help reduce lung cancer mortality.

Inflammation is a physiological response of the innate immune system to infection and tissue injury. It aims to deliver and activate leucocytes to the site of interest where they will be in charge of fighting the external agent, notably through neutrophil degranulation which releases unspecific toxic agents such as reactive oxygen species [3]. In physiological conditions, inflammation is an acute and self-limiting process that will resolve itself once the initial problem is eliminated. In other conditions, however, inflammation can become chronic and be involved in several health impairments, including type 2 diabetes, atherosclerosis, asthma, neurodegenerative diseases, rheumatoid arthritis and cancer [3,4]. The paradigm of inflammation-driven carcinogenesis started more than 150 years ago and is now widely accepted, with practical examples such as hepatitis-associated liver cancer, *Helicobacter pylori*-induced stomach cancer or colitis-associated colon cancer. Consistently, members of the nuclear factor kappa B (NF-κB) family, which are key regulators of inflammation, have been shown to be overactivated in most tumors, making them some of the main actors linking inflammation to cancer [5].

The NF-κB family is composed of five proteins (RelA or p65, RelB, c-REL, p50 and p52). Various dimer combinations have been shown to regulate the expression of over 500 genes. Dimers are generally kept in the cytoplasm by repressors and their activation depends on two principal routes—the classical and the alternative—involving various actors with different outcomes. While the first one is involved in the activation of innate immunity and inflammation, the latter is important for adaptive humoral immunity, maturation of B cells and secondary lymphoid organ development [6]. In the classical pathway, p50/p65 dimers are bound to the NF-κB inhibitor α (IκBα), forcing them to remain in an inactive state in the cytoplasm. Upon stimuli (TNF-α, LPS, genotoxic agents), IκBα kinase (IKK) is activated, which leads to IκBα phosphorylation, ubiquitination, and proteasomal degradation. This allows nuclear translocation of the released p50/p65 complex and induction of target genes transcription. Besides its role in inflammation, the classical NF-κB pathway also regulates multiple aspects of carcinogenesis such as cell proliferation, survival, invasion, angiogenesis, and metastasis [5]. Additionally, chemotherapy-induced NF-κB activation is thought to drive chemoresistance [5]. Several articles reported a crucial role for overactivation of the NF-κB pathway in lung carcinogenesis. Among others, Tang et al. described significantly higher levels of NF-κB activity in human lung cancers than in healthy tissues [7], and Takahashi et al. showed that tobacco smoke-induced lung carcinogenesis involved NF-κB-dependent inflammation in mice [8]. Therefore, potent NF-κB inhibitors present a high interest for cancer chemoprevention as well as therapy. In 2006, Gilmore and Herscovitch counted more than 785 NF-κB inhibitors acting at various steps of the pathway, almost half of them coming from natural sources [9]. Among those natural sources, fungi represent a highly interesting field to explore with only a few NF-κB inhibitors having been described as of today. In our search for chemopreventive drugs from natural sources, the ethyl acetate (EtOAc) extract of *Penicillium vulpinum* (Cooke and Massee) Seifert and Samson was found to potently inhibit NF-κB activity. Patulin was identified as the main NF-κB inhibitor in this mycotoxigenic fungus, and its potential interest for lung cancer chemoprevention was investigated. This study shows for the first time the proapoptotic, antimigratory and Wnt inhibitory activities of patulin in lung adenocarcinoma cells.

## 2. Results

### 2.1. Patulin Isolation through Bioassay-Guided Fractionation

**Figure 1:**
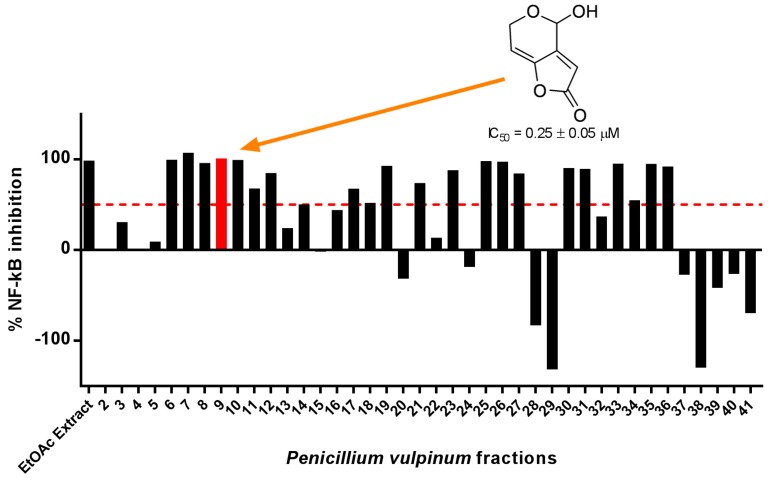
Cultured *P. vulpinum* was extracted in ethyl acetate (EtOAc) and fractionated by flash chromatography using two C18 columns and a linear gradient of H_2_O + formic acid (FA) 0.1% and acetonitrile (ACN) + FA 0.1% from 98:2 to 2:98 at 14 mL/min for 200 min. Fractions were tested for their ability to inhibit TNF-α-induced NF-κB activity in HEK293 cells at 20 μg/mL. Fractions able to inhibit more than 50% of NF-κB activity were considered active. F9 was the most interesting in terms of activity and available amount. Patulin was isolated from this fraction and inhibited NF-κB with an IC_50_ value of 0.25 ± 0.05 µM.

The screening of several extracts against NF-κB inhibition identified the crude EtOAc extract of *P. vulpinum*, which inhibited TNF-α-induced NF-κB activity by 99% at 20 μg/mL, and was therefore selected for bioguided fractionation. Among the many fractions that substantially inhibited NF-κB, F9 was selected based on activity, available amounts, and simplicity of the chromatographic profile. The major compound of this fraction was further purified and identified as patulin by comparison of its spectral data (High-Resolution Mass Spectrometry (HRMS) and NMR) to those reported in the literature [10]. The IC_50_ value for NF-κB inhibition of patulin was 0.25 μM (Figure 1 and Figure S1), making worthy of investigation its mechanism of action and its effect on other important aspects of lung carcinogenesis, such as cell viability and migration. These further investigations were performed using a human lung adenocarcinoma model (A549 cell line), which is the most frequent form of lung cancers where induction of NF-κB is thought to contribute to tumor aggressiveness [11].

### 2.2. Patulin Inhibited NF-κB p65 Nuclear Translocation

**Figure 2:**
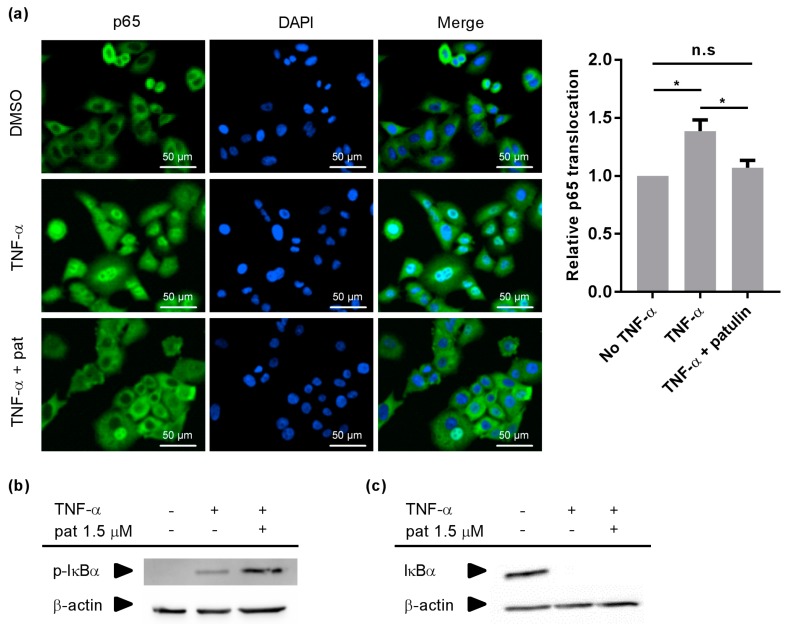
Patulin inhibited TNF-α-induced p65 nuclear translocation without acting on IκBα phosphorylation or degradation. (**a**) NF-κB p65 immunocytochemical translocation pattern in A549 cells following 30 min incubation with either DMSO, 20 ng/mL TNF-α or TNF-α + 1.5 μM patulin (Pat). DAPI counterstaining (0.1 μg/mL) was used to confirm nuclear localization. Quantification of relative p65 nuclear translocation was performed from the immunocytochemistry pictures (*n* = 3). * *p* < 0.05; (**b**) Western blot analysis showing the effect of patulin (1.5 μM) on TNF-α-induced IκBα phosphorylation after 5 min. The experiment was repeated twice and similar results were obtained in both cases; (**c**) Western blot analysis showing the effect of patulin (1.5 μM) on TNF-α-induced IκBα after 15 min. The experiment was repeated three times and similar results were obtained.

To better understand how patulin affected the NF-κB pathway, its effect on TNF-α-induced p65 nuclear translocation was investigated in A549 cells using immunocytochemistry, with a concentration of patulin (1.5 μM) that inhibited roughly 90% of NF-κB activity in HEK cells , which was selected to allow a clear visualisation of the inhibitory effect. Patulin suppressed TNF-α-induced p65 nuclear translocation (Figure 2a), indicating that its target was a cytoplasmic step of the NF-κB activation pathway. Having ruled out a nuclear mechanism of action, investigations were focused on IKK, the most commonly described cytoplasmic target for NF-κB inhibitors. The effect of patulin treatment on TNF-α-induced IκBα phosphorylation was therefore evaluated using Western blots. Patulin had no impact on IκBα phosphorylation (Figure 2b), indicating that its action was independent from IKK activity. The target of patulin in the NF-κB activation cascade should therefore happen between IκBα phosphorylation and p65 nuclear translocation. The last important steps that needed to be investigated were IκBα ubiquitination and proteasomal degradation. Patulin did not impact TNF-α-induced IκBα degradation (Figure 2c), pointing towards nuclear translocation as the most probable target of patulin.

### 2.3. Patulin Triggered Apoptotic Cell Death in A549 Cells

**Figure 3:**
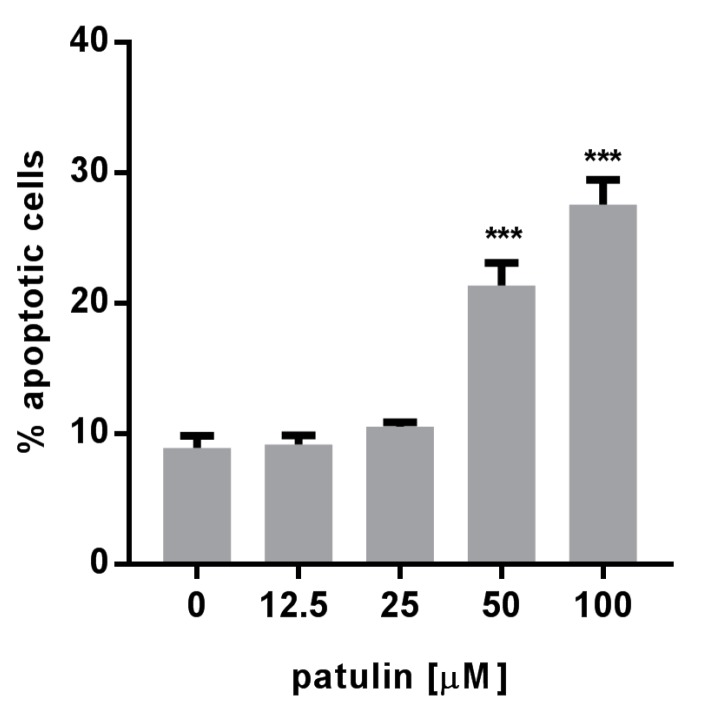
Dose-dependent increase in percentage of both early and late apoptosis 48 h after patulin treatment, evaluated by the measurement of all annexin V positive cells by flow cytometry (*n* = 3). *** *p* < 0.001.

Considering the implication of the NF-κB pathway in cancer cell proliferation and survival, the cytotoxicity of patulin was measured in A549 cells using the well-established sulforhodamine B (SRB) assay. Patulin exhibited dose-dependent cytotoxicity in those cells with an IC_50_ of 8.8 µM (Figure S2). A significant dose-dependent increase in the percentage of annexin V-marked cells after patulin treatment pointed towards apoptosis as a possible mechanism involved in the observed cell death (Figure 3).

### 2.4. Patulin Inhibited A549 Cell Migration

**Figure 4:**
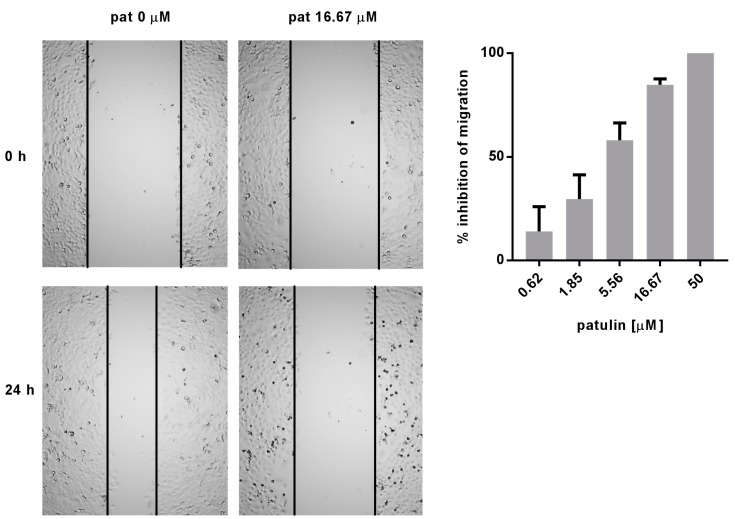
Dose-dependent inhibition of A549 cells migration 24 h after patulin treatment, evaluated through the scratch assay. Time-lapse videos are available as supplementary material. Quantification of migration inhibition was performed on three independent experiments.

Cell migration represents another important aspect of carcinogenesis regulated by the NF-κB pathway. The effect of patulin on cell migration was assessed through the wound healing (scratch) assay. Patulin dose-dependently inhibited cell migration, with an estimated IC_50_ below 5.5 μM (Figure 4). Time-lapse videos were recorded for 24 h to make sure that the free space recovery was due to migration inhibition and not an anti-proliferative effect (Video S1 and S2).

### 2.5. Patulin Inhibited the Wnt Pathway

**Figure 5:**
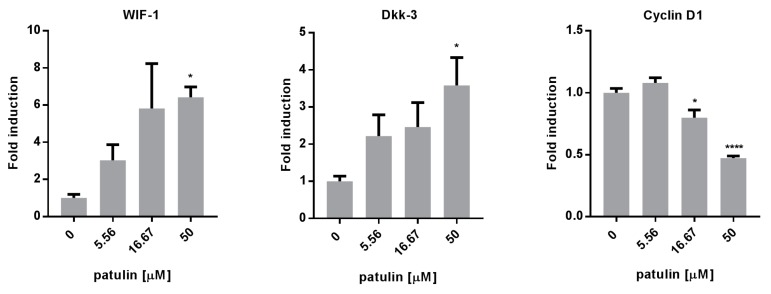
Patulin inhibited the Wnt pathway. RT-qPCR analysis showed that a 24 h treatment with patulin upregulated the expression of WIF-1 and Dkk-3, two endogenous inhibitors of the Wnt pathway, and downregulated Cyclin D1, a target gene of this pathway. * *p* < 0.05, **** *p* < 0.0001.

To better understand which other pathways, beside NF-κB, could be involved in the proapoptotic and antimigration activity of patulin, the expressions of several genes involved in both mechanisms were measured using quantitative real time PCR in A549 cells 24 h after treatment with increasing doses of patulin. The expression of Wnt inhibitory factor-1 (WIF-1) and Dickkopf-related protein 3 (Dkk-3) (Figure 5), two endogenous inhibitors of the Wnt pathway, which are frequently downregulated in lung cancer, were upregulated. This correlated with the downregulation of Cyclin D1 expression, a target gene regulated by the Wnt pathway.

## 3. Discussion

Patulin is a well-known mycotoxin produced by several fungi of the *Aspergillus* and *Penicillium* genera [12]. It is considered as a contaminant in apples and apple-derived products. Beside the numerous articles regarding the food safety concern it represents, recent work reported that patulin also possessed potent anticancer activity through apoptosis induction in cancer cell lines and in one in vivo model of melanoma cells-bearing mice [13,14]. The present study shed light on its lung cancer chemoprevention properties by studying its inhibitory activity on TNF-α-induced NF-κB activity, its proapoptotic and antimigration activity on A549 cells, as well as its ability to inhibit the Wnt pathway.

The NF-κB pathway is a complex cascade involving several steps that can each be the target of inhibitors. The majority of natural inhibitors act through the inhibition of IKK, NF-κB DNA binding, or IκBα proteasomal degradation [9]. In the present study, observations of p65 translocation inhibition by patulin led the list of potential targets being restricted to the cytoplasmic steps of the pathway. Absence of changes in both TNF-α-induced IκBα phosphorylation and degradation indicated that patulin’s probable mechanism of action was a direct inhibition of p65 translocation. To our knowledge, this work presents the first report of the inhibitory activity of patulin against TNF-α-induced NF-κB activity. Interestingly, Tsai et al. recently described NF-κB inhibitory properties of patulin that seemed specific to LPS induction [15]. Consistent with the present work, they observed no effect on TNF-α-induced IκBα degradation after patulin treatment. This was, however, interpreted as an inactivity of patulin on TNF-α-induced NF-κB activity, a hypothesis that should be reformulated in regard to the present observations. In the search for NF-κB inhibitors, IKK represented the main target of the pharmaceutical industry, owing to the expected specificity of its inhibitors as compared with proteasome or ubiquitination inhibitors [6]. However, recent findings such as IκBα-independent NF-κB activation through NFKBIA (the gene coding for IκBα) deletion in most glioblastomas [16] or resistance to proteasome inhibitors in in vivo models [17] highlight the potential interest of inhibitors that act downstream of the IkB phosphorylation/degradation process. Indeed, IKK and proteasome inhibitors would become ineffective in the case of a defective IκB production. Therefore, inhibitors of p65 nuclear translocation, such as patulin, could present a particular interest by being both specific and less prone to chemoresistance.

In addition to its NF-κB inhibitory properties, patulin showed antiproliferative, proapoptotic and antimigration activities. Each of these activities could at least be partly explained by patulin’s ability to inhibit the Wnt pathway. Overactivation of the Wnt pathway through downregulation of its inhibitors is indeed common in lung cancer and has been associated with poor prognosis, chemoresistance and metastasis [18,19]. Consistently, inhibition of this pathway, for example through restoration of endogenous inhibitors such as WIF-1 and Dkk-3 resulted in tumor regression, increased apoptosis and reduced cell motility both in vitro and in vivo [18,20,21]. Interestingly, cigarette smoke extract was shown to induce the Wnt pathway in normal human bronchial epithelial cells, contributing to carcinogenesis through the upregulation of target genes such as Cyclin D1 [22]. Therefore, inhibitors of the Wnt pathway are particularly interesting for lung cancer chemoprevention and therapy, and clinical trials are being conducted for this purpose [23,24].

Altogether, these data support the interest for patulin in lung cancer chemoprevention and therapy, and should encourage the realization of further in vivo experiments, using, for example, chemically-induced lung carcinogenesis models closely mimicking tobacco-induced lung cancer in humans. Although previous in vivo experiments have not reported side effects upon treatment with active doses of patulin [13], one should remain aware of its toxicity [25] and adapt the doses consequently. Further investigations on patulin derivatives (e.g., precursors) could lead to the discovery of interesting active compounds with less toxicity.

## 4. Materials and Methods

### 4.1. Fungal Culture

The strain of *Penicillium vulpinum* (Cooke and Massee) Seifert and Samson was isolated in May 1999 in Lausanne (VD, Switzerland) on mature tomatoes stored at 4 °C and authenticated by molecular sequencing of the ITS region. The strain is maintained and stored (n° 932) in the Mycoscope dynamic mycotheca of Agroscope (www.mycoscope.bcis.ch) in Potato Dextrose Broth (PDB). *P. vulpinum* was cultivated in 74 glass bottles each containing 100 mL of PDB media supplemented with 100 µM suberoyl anilide hydroxamic acid (SAHA, Sigma-Aldrich, Saint-Louis, MO, USA). *P. vulpinum* was allowed to grow in static mode, for 15 days with artificial day/night alternation (12 h each) at 22 °C.

### 4.2. Extraction and Isolation of Patulin

The mycelial mat was filtered on Büchner and the culture media was extracted by liquid/liquid partition with equal volume of EtOAc. The extraction process was repeated three times. Reduced pressure evaporation of the EtOAc fraction afforded 6.9 g of crude extract (brown liquid gum). Vacuum Liquid Chromatography (VLC) filtration on 70 g of C18 silica and elution with methanol (MeOH):water (H_2_O) (90:10) afforded 6.5 g of extract. This extract was fractionated by flash chromatography on a Puriflash system (Interchim, Montluçon, France). Two 120 g columns of C18 were connected and the crude extract was dry-loaded using celite. A linear gradient of H_2_O + formic acid (FA) 0.1% and acetonitrile (ACN) + FA 0.1% from 98:2 to 2:98 was applied at 14 mL/min for 200 min and 264 fractions were collected. An aliquot (200 µL) of each fraction was sampled and plated into 96-well plates for analysis. The rest of the fractions were dried using a centrifugal evaporator (Genevac HT-4, SP Scientific, Gardiner, NY, USA). After UHPLC-MS/UV/ELSD analysis, fractions were further pooled into 41 fractions (F1-F41) and tested against NF-κB. F9 was selected based on bioactivity profile. F9 (130 mg) was loaded on an Armen LC system using a preparative Kinetex Axia Core-Shell C18 column (5 μm, 250 × 21.2 mm; Phenomenex, Torrance, CA, USA) with elution in isocratic mode (H_2_O + 0.1% FA:MeOH + 0.1% FA) at 20 mL/min and afforded 56.2 mg of a compound, which was identified as patulin (purity > 95%) based on comparison of its experimental NMR and HRMS spectra with the literature [10].

### 4.3. NMR and HRMS Measurements

The NMR spectroscopic data were recorded on a Bruker Avance III HD 600 MHz NMR spectrometer equipped with a QCI 5 mm Cryoprobe and a SampleJet automated sample changer (Bruker BioSpin, Rheinstetten, Germany). The NMR spectra were recorded in DMSO-*d6*. High-resolution mass spectrometric date were recorded on a Q-Exactive Plus mass spectrometer (Thermo Fisher scientific, Waltham, MA, USA) interfaced to a Thermo Dionex Ultimate 3000 UHPLC system, using a heated electrospray ionization (HESI-II) source. Full scans were acquired at a resolution of 35,000 FWHM (at *m*/*z* 200) and MS/MS scans at 17,500 FWHM both with a maximum injection time of 50 ms.

### 4.4. Cell Culture

HEK293/NF-κB-luc cells (Panomics, Fremont, CA, USA) were cultured at 37 °C and 5% CO_2_ atmosphere in Dulbecco’s modified Eagle’s medium (Thermo Fisher Scientific) with 10% fetal bovine serum (Biowest, Nuaillé, France), 100 U/mL penicillin, 100 μg/mL streptomycin (Thermo Fisher scientific) and 100 μg/mL hygromycin B (Thermo Fisher scientific). A549 cells (ATCC, Manassas, VA, USA) were cultured at 37 °C and in a 5% CO_2_ atmosphere in F-12K medium (Thermo Fisher scientific) with 10% fetal bovine serum (Biowest), 100 U/mL penicillin and 100 μg/mL streptomycin (Thermo Fisher scientific).

### 4.5. Measure of NF-κB Activity

NF-κB inhibitory activity was assessed using a HEK293/NF-κB-luc cell line as previously described [26]. Briefly, cells were incubated 1 h in FBS-free medium with 2.5 µM of Cell Tracker Green CMFDA (Thermo Fisher Scientific), a fluorescent dye used to measure cell viability, and seeded in 96-well plates (10^4^ cells/well). After an overnight incubation, cells were treated with patulin or vehicle only (0.5% DMSO in culture medium) and stimulated with TNF-α (20 ng/mL) (Sigma-Aldrich) for 5 h. Then, the cells were lysed with reporter lysis buffer (Promega, Madison, WI, USA) and both fluorescence of the Cell Tracker Green CMFDA and luminescence of the firefly luciferase were read on a Cytation 3 imaging multimode reader (Biotek, Winooski, VT, USA). The luminescence signal was normalized by the fluorescence signal for each well, and relative NF-κB activity was quantified by comparing the normalized luminescence signal of sample-treated cells with the vehicle-treated cells. Nonlinear regression (with sigmoidal dose response) was used to calculate the IC_50_ values using GraphPad Prism 6.05. Each compound was tested in duplicate and three independent experiments were performed. Parthenolide (Tocris Bioscience, Bristol, UK) was used as a positive control. Extracts and fractions were screened at 20 μg/mL. Patulin dose–response curve started at 10 μM.

### 4.6. Immunocytochemistry

A549 cells were seeded in clear-bottom black 96-well plates (Corning, New York, NY, USA) (10^4^ cells/well). After overnight incubation, cells were treated with either a vehicle control (0.5% DMSO in culture medium), TNF-α only (20 ng/mL), or 1.5 µM patulin + TNF-α (20 ng/mL) for 20 min. Cells were then rinsed with DPBS (Thermo Fisher scientific), fixed with 4% paraformaldehyde for 10 min, and permeabilized 5 min with a 0.1% Triton X-100 DPBS solution (DPBST). Blocking was then performed for 30 min with 1% BSA in DPBST, and cells were incubated overnight at 4 °C with the rabbit anti-p65 antibody (Cell Signaling Technology, Danvers, MA, USA). After rinsing three times with DPBS, cells were incubated for 1 h at room temperature in the dark with an anti-rabbit antibody (Cell Signaling Technology), and counterstained with 0.1 µg/mL DAPI for 1 min. After rinsing three times in DPBS, fluorescent pictures were taken on the Cytation 3 imaging multimode reader (Biotek). NF-κB p65 nuclear translocation was quantified by measuring the fluorescence intensity of the secondary antibody in the nuclear zones defined by DAPI staining, using the Gen5 software 3.0 (Biotek).

### 4.7. Western Blot Analysis

A549 cells were seeded in 10 mm Petri dishes (2.2 × 10^6^ cells/dish) and grown for 48 h until 80–90% confluency. Cells were then treated with either vehicle control (0.5% DMSO in culture medium), TNF-α only (20 ng/mL), or 1.5 µM patulin + TNF-α (20 ng/mL), during 5 min for observation of IκBα phosphorylation and 15 min for IκBα degradation. Cells were then rinsed, harvested with TryplE Express (Thermo Fisher Scientific), and cytoplasmic extraction was performed using the NE-PER extraction kit (Thermo Fisher Scientific). Protein concentration of each cytoplasmic extract was determined on a Qubit 3.0 fluorometer (Thermo Fisher Scientific). After protein denaturation, 20 µg of proteins were loaded on 12.5% bis-acrylamide gels, and electrophoresis was performed at 100 V. Proteins were then transferred to a PVDF membrane for 40 min at 10 V. After the transfer, membranes were blocked in 5% non-fat milk for 60 min under agitation, and incubated overnight with the primary antibodies (1:1000 in 1% non-fat milk), either mouse anti-IκBα or rabbit anti-phospho-IκBα (Cell Signaling Technology) at 4 °C. After rinsing three times with wash buffer, membranes were incubated with the corresponding anti-mouse or anti-rabbit horseradish peroxidase-conjugated secondary antibody (Cell Signaling Technology) for 1 h at room temperature, followed by detection using SuperSignal™ West Pico Chemiluminescent Substrate (Thermo Fisher Scientific) on a myECL imager (Thermo Fisher Scientific).

### 4.8. Cell Viability Assay

A549 cells were seeded in clear 96-well plates (Corning) (10^4^ cells/well). After overnight incubation, cells were treated with 0.5% DMSO or increasing doses of patulin for 72 h. Cells were then fixed with cold trichloroacetic acid for 30 min, rinsed with tap water and dried overnight at room temperature. Proteins were stained with a 0.4% SRB solution for 30 min and cells were rinsed four times with a 1% acetic acid solution. The bound SRB was then solubilized in a 10 mM Tris base solution and absorbance was read at 510 nm using a Cytation 3 imaging multimode reader. Nonlinear regression (with sigmoidal dose response) was used to calculate the IC_50_ values using GraphPad Prism 6.05.

### 4.9. Apoptosis Assay

Apoptosis was measured using the annexin V-fluorescein isothiocyanate (V-FITC)/propidium iodide (PI) assay according to the manufacturer’s protocol (Thermo Fisher Scientific). A549 cells were seeded in a 12 wells plate (10^5^ cells/well). After overnight incubation at 37 °C in a 5% CO_2_ atmosphere, cells were treated with 0.5% DMSO or increasing doses of patulin for 48 h. Detached cells were collected with the medium, while attached cells were trypsinized. All recovered cells were rinsed with PBS and stained with 5 µL annexin V-FITC and 1 µL PI (100 µg/mL) for 15 min in the dark at room temperature. Percentage of all apoptotic cells was determined by counting the percentage of annexin V-FITC positive cells using an attune NxT flow cytometer (Thermo Fisher Scientific).

### 4.10. Cell Migration Assay

The scratch assay was used to determine cell migration as previously described [27]. Briefly, A549 cells were seeded in a 96-well plate (1.5 × 10^4^ cells/well) and grown until confluence. A scratch was then drawn in each well using a 200 µL pipette tip and debris were removed by rinsing cells with DPBS. Cells were then treated with increasing concentrations of patulin or with DMSO alone (0.5% max). Pictures were taken quickly after treatment and after 24 h of incubation at 37 °C in a 5% CO_2_ atmosphere. Widths of the wounds were measured at both time points using the Gen5 software 3.0 (Biotek) and percentage of space recovery after 24 h was obtained using the formula: % recovery = 100 − (width 24 h/width 0 h × 100). Inhibition of cell migration was quantified by comparing the percentage of space recovery between the cells treated with patulin and the cells treated with DMSO alone. 

### 4.11. Quantitative Real Time PCR Analysis of mRNA Expression

A549 cells were seeded in 6-well plates (3.5 × 10^5^ cells/well) and allowed to adhere overnight before treatment with increasing doses of patulin. After a 24 h treatment, cells were lysed, and total RNA was isolated using the Aurum total RNA Mini Kit (Bio-Rad, Cressier, Switzerland). The “high capacity total RNA to cDNA” Reverse Transcriptase (Thermo Fisher scientific) and a PCR standard thermal cycler (Thermo Fisher scientific) were used to perform the reverse transcription of 0.25 μg RNA. One microliter cDNA was amplified by quantitative PCR using a SYBR Green PCR Kit (Thermo Fisher scientific) and a Step One Plus Real-Time PCR Thermal Cycler (Thermo Fisher scientific). Custom primers for *GAPDH*, *WIF1*, *DKK3*, and *CCND1* were designed on http://bioinfo.ut.ee/primer3-0.4.0/ and obtained from Thermo Fisher scientific [28,29]. Relative gene expression was calculated by normalizing to the housekeeping *GAPDH* using the ΔΔ*C*_T_ method.

### 4.12. Statistical Analysis

Results are presented as means ± standard errors. Relative p65 nuclear translocation was compared using one-way ANOVA followed by Tukey’s multiple comparison test. Percentages of apoptotic cells as well as relative gene expression were compared to the DMSO control using a one-way ANOVA followed by Dunnett’s multiple comparison test. A *p* value < 0.05 was considered significant.

## Supplementary Materials

Supplementary materials are available online. Video S1: A549 cell migration for 24 h without patulin. Video S2: A549 cell migration for 24 h with 16.67 µM patulin. Dose–response curves for NF-κB inhibition and A549 cell viability are presented in Figures S1 and S2, respectively. Results of the NMR experiments are presented in Figures S3–S7.
