# Supplementary material for: Lung Cancer Chemopreventive Activity of Patulin Isolated from Penicillium vulpinum
Source: Molecules. 2018 Mar 12;23(3):636. doi: 10.3390/molecules23030636 (PMC6017800; doi:10.3390/molecules23030636)
Supplement: Supplementary file 1 [file molecules-23-00636-s001.zip › Patulin Supplementary_revised2.docx]

Supplementary material

Lung cancer chemopreventive activity of patulin isolated from *Penicillium vulpinum*

Aymeric Monteillier ^1^, Pierre-Marie Allard ^1^, Katia Gindro ^2^, Jean-Luc Wolfender ^1^ and Muriel Cuendet ^1,^*

^1^ School of pharmaceutical sciences, University of Geneva, University of Lausanne, 1 Rue Michel-Servet, CH-1211 Geneva 4, Switzerland

^2^ Mycology and Biotechnology group, Plant Production, Agroscope, Route de Duillier 60, P.O. Box 1012, 1260 Nyon, Switzerland

***** Correspondence: muriel.cuendet@unige.ch; Tel.: +41 22 379 33 86

**
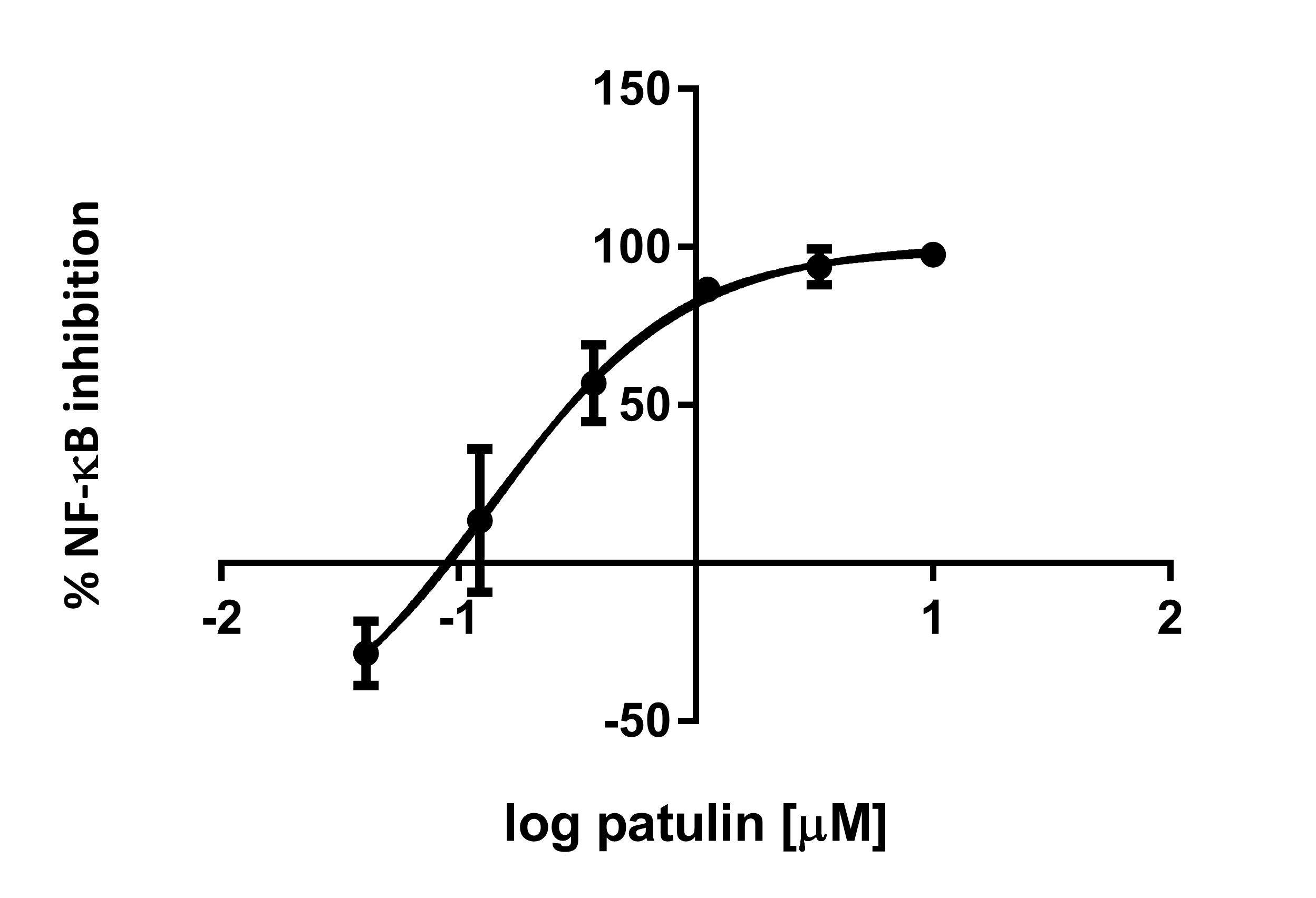
**

**Figure S1:** Dose response curve for NF-κB inhibition in HEK293 cells

**
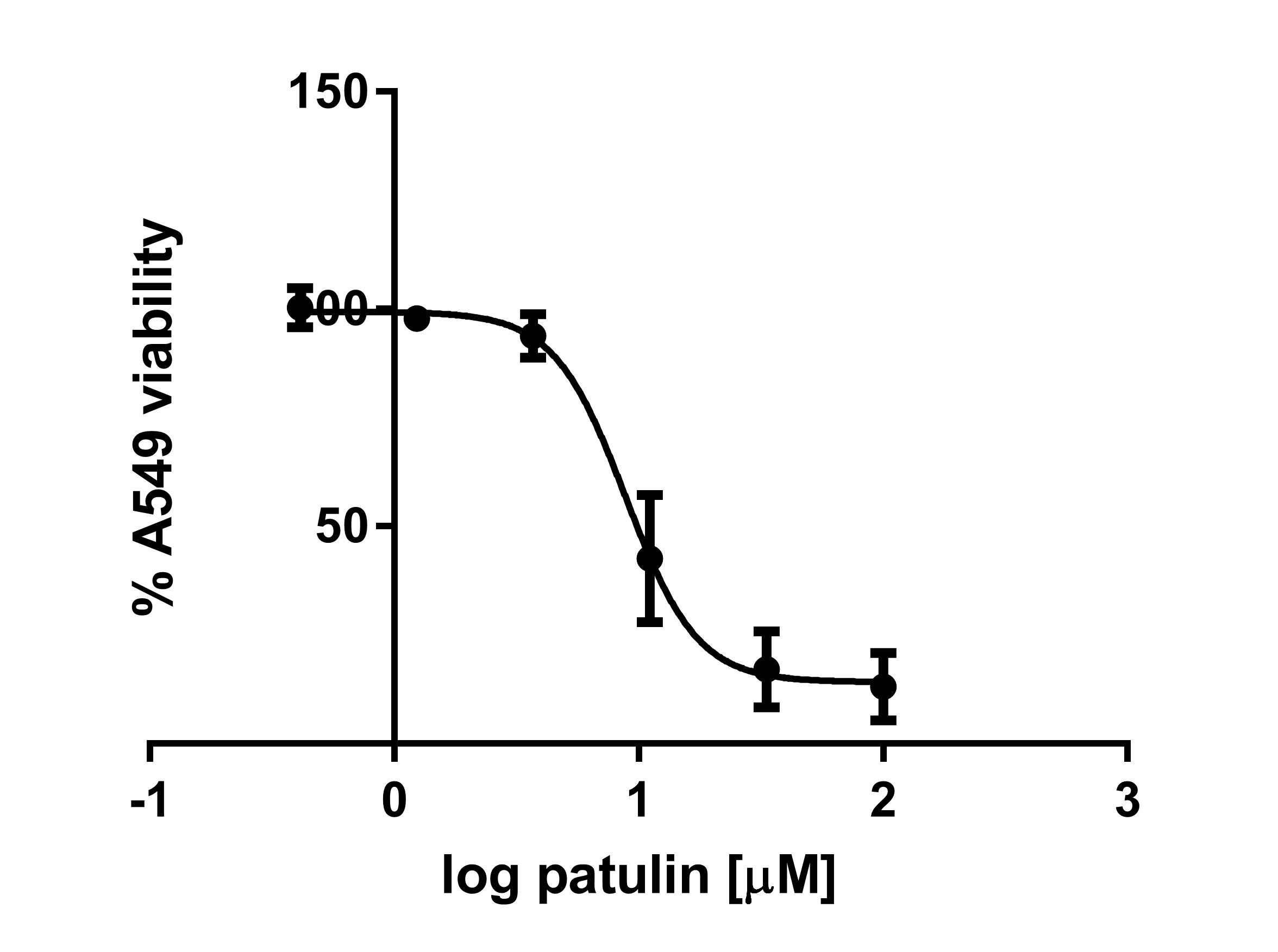
**

**Figure S2:** Dose response curve for A549 cell viability


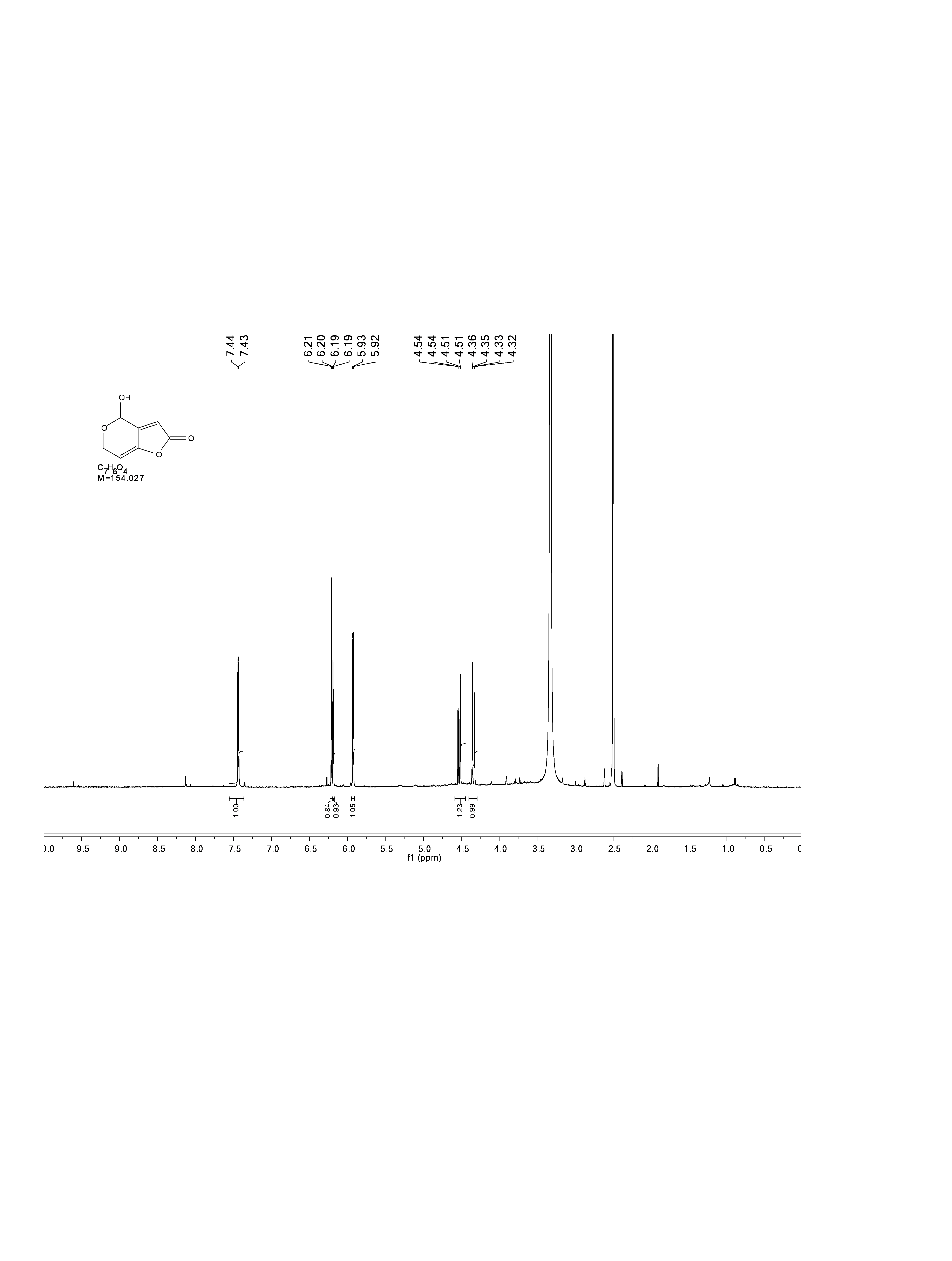


**Figure S3**: ^1^H NMR spectrum

**
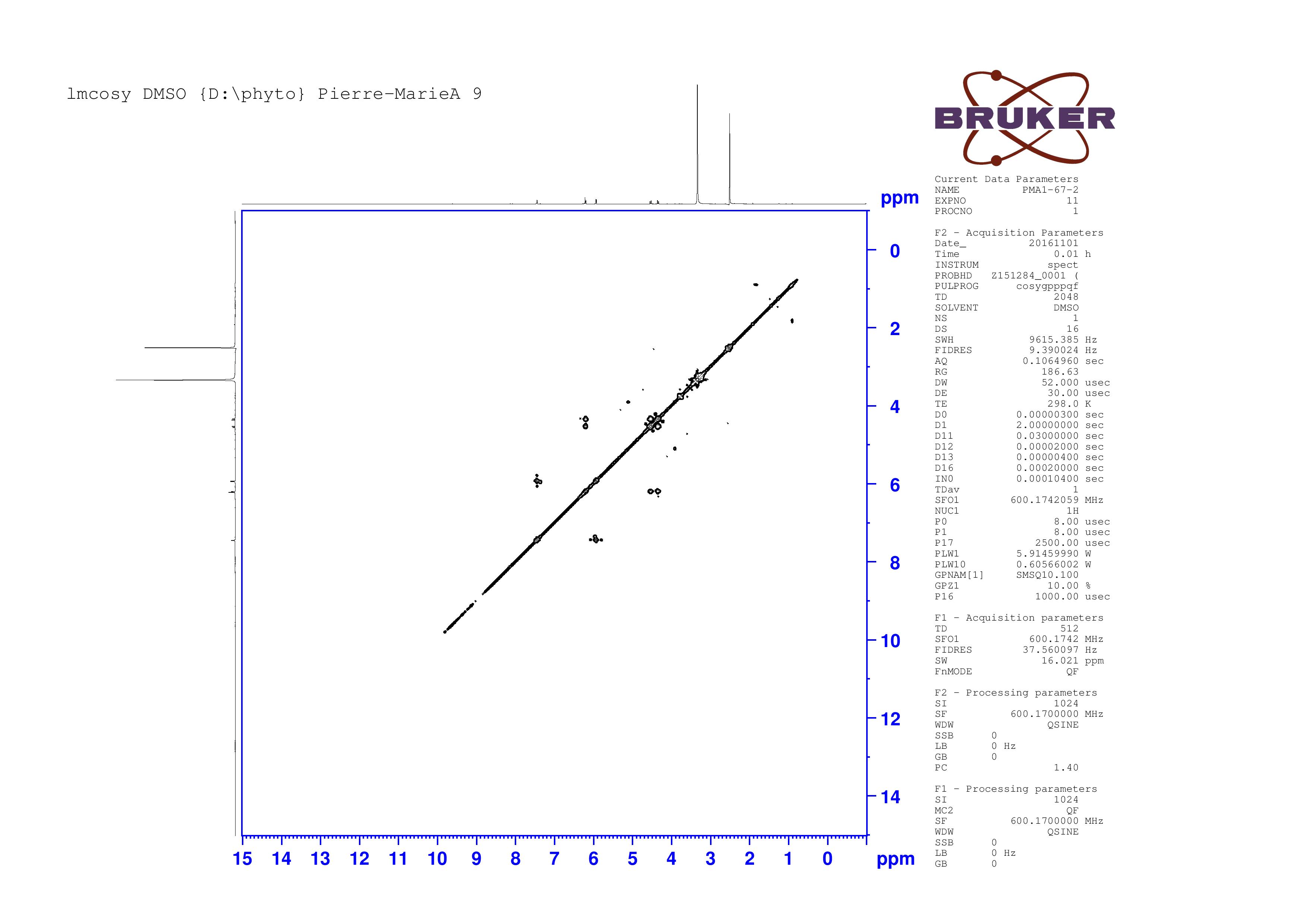
**

**Figure S4:** COSY spectrum

**
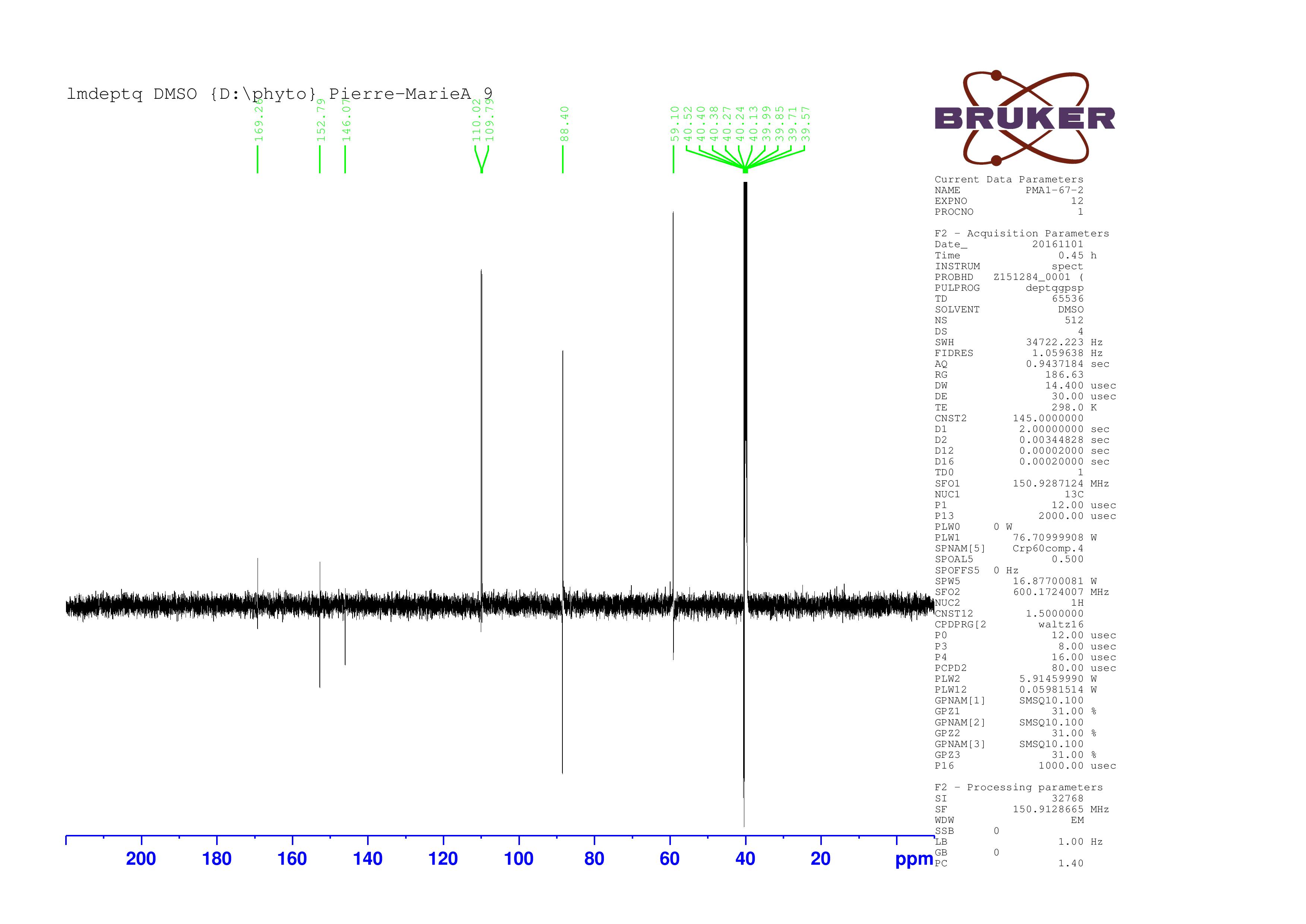
**

**Figure S5:** DEPT spectrum

**
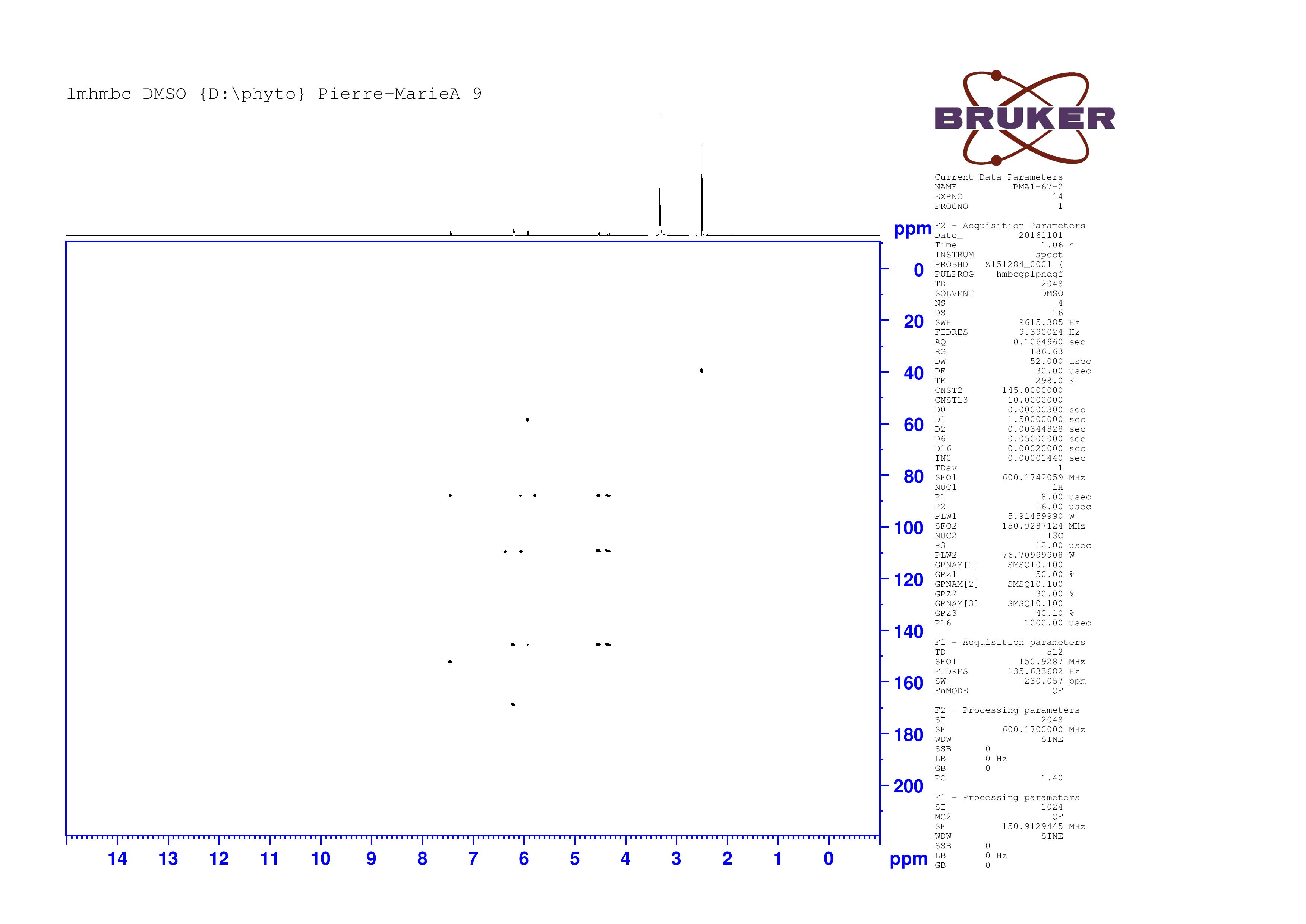
**

**Figure S6:** HMBC spectrum

**
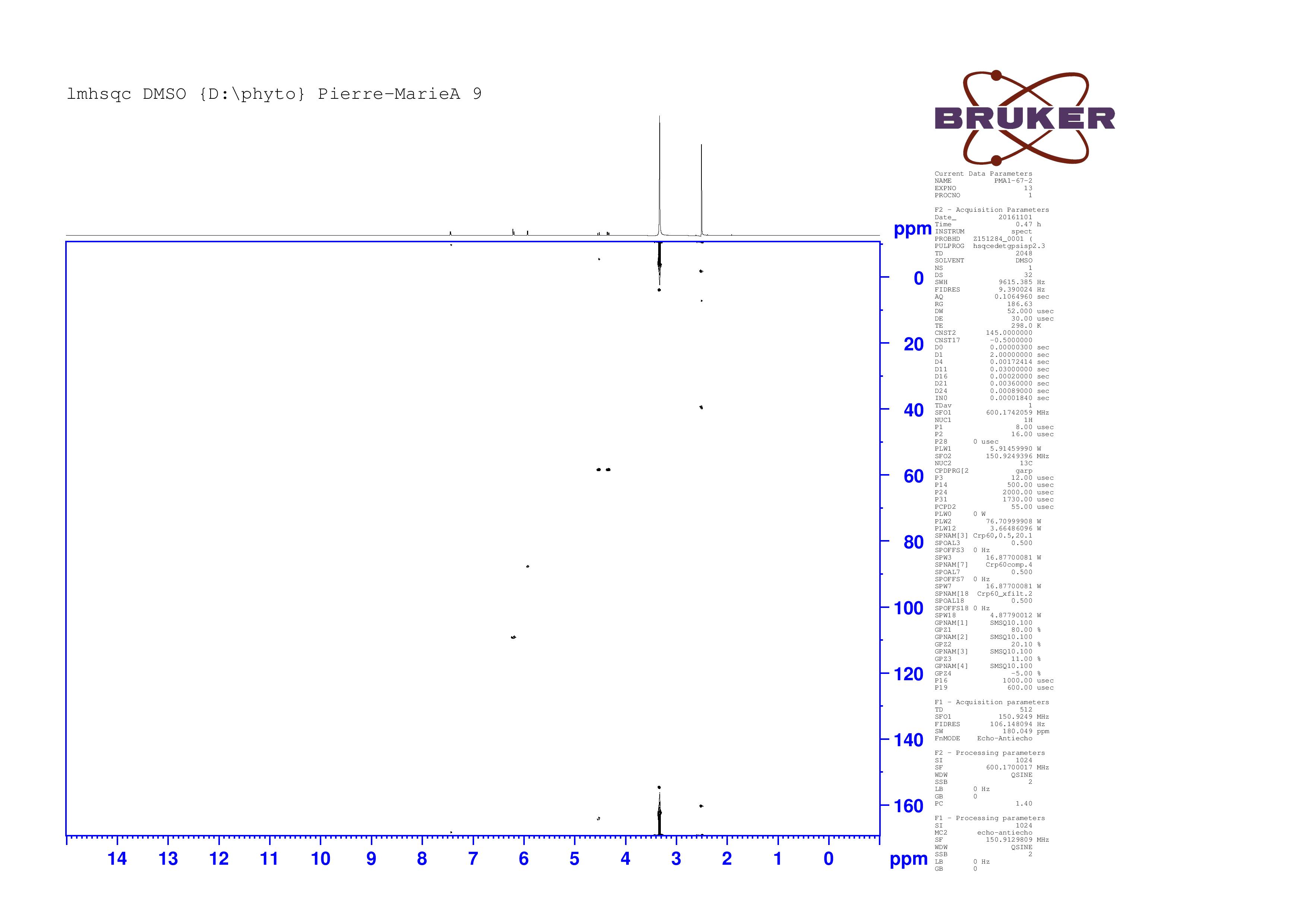
**

**Figure S7:** HSQC spectrum
